# Ash Interaction with Two Cu-Based Magnetic Oxygen Carriers during Biomass Combustion by the Chemical Looping with Oxygen Uncoupling Process

**DOI:** 10.1021/acs.energyfuels.4c02464

**Published:** 2024-10-04

**Authors:** A. Filsouf, I. Adánez-Rubio, T. Mendiara, A. Abad, J. Adánez

**Affiliations:** Carboquimica Institute, Miguel Luesma Castán, 4, 50018 Zaragoza, Spain

## Abstract

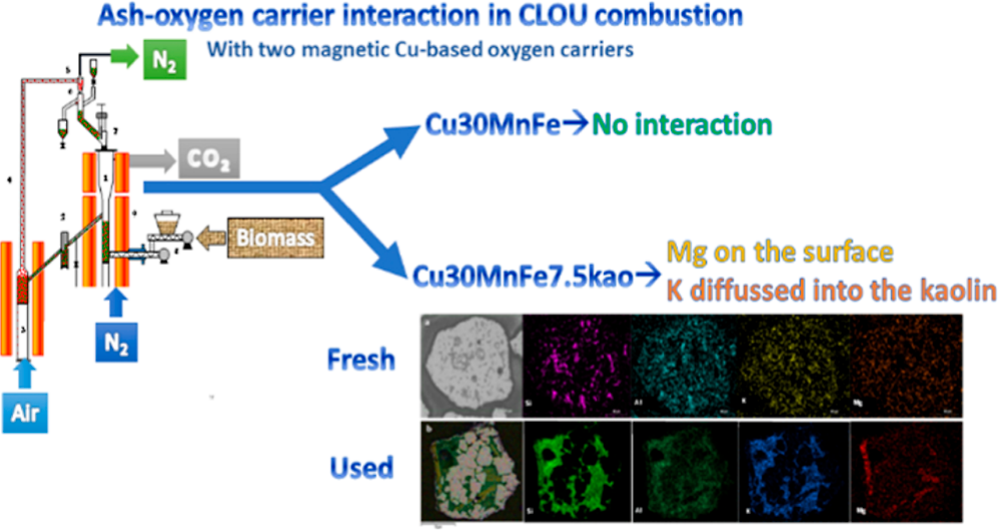

Chemical-looping combustion (CLC) stands out as a promising method for carbon capture and storage for the purpose of mitigating climate change. The process involves the conversion of fuel facilitated by an oxygen carrier, with the resulting CO_2_ inherently separated from other air components. Notably, when applied to biomass combustion this process offers a pathway to achieving negative CO_2_ emissions. However, a significant challenge for CLC, particularly in its application to biomass, is the management of interactions between ash and oxygen carriers. Biomass-derived ashes typically contain substantial quantities of reactive ash-forming substances, such as alkaline and alkali earth elements. These interactions can impact the performance and longevity of the oxygen carrier, necessitating careful consideration and mitigation strategies in CLC systems utilizing biomass feedstocks. This study examined the interaction between biomass ash components and two recently developed oxygen carriers, Cu30MnFekao7.5 and Cu30MnFe, during combustion in a 1.5 kW_th_ continuous unit. Both oxygen carriers achieved 100% combustion efficiency and a CO_2_ capture efficiency of 95% at 900 °C. Although the copper in both oxygen carriers did not exhibit any noticeable interaction with ash components, the accumulative presence of potassium and magnesium in Cu30MnFekao7.5 was identified by inductively coupled plasma and scanning electron microscopy with energy dispersive X-ray analysis, indicating an increase in the amount of both elements in the particles after combustion operation. No problems of agglomeration or fluidization were observed in any of the experiments.

## 1 Introduction

The goal of limiting global warming to below 2 °C has led to the setting of strict limits on carbon emissions,^1^ although these are highly likely to be exceeded considering the ongoing rise in CO_2_ emissions, with a current value of 425 ppm reported in March 2024.^2^ To adhere to the Paris agreement plan, and particularly the more stringent target of keeping warming below 1.5 °C, the implementation of carbon dioxide removal technologies from the atmosphere becomes evidently necessary.^3^ Numerous negative emission technologies have been suggested, among which bioenergy with carbon capture and storage (BECCS) is gradually emerging as a highly promising option, demonstrating considerable potential in terms of its cost-effectiveness and capacity to remove carbon from the atmosphere.^4,5^ Chemical-looping combustion (CLC) stands out as a promising technology that enables the separation of CO_2_ from the combustion process at relatively low cost and with a low efficiency penalty.^6^ In CLC, the oxygen needed for the fuel combustion is supplied by a solid oxygen carrier (OC), therefore avoiding gas mixing. The oxygen carrier, typically a metal oxide, circulates between two interconnected reactors: the fuel and air reactors. The fuel reactor (FR) is where the fuel is burned to CO_2_ and H_2_O, causing the oxygen carrier to be reduced. The reduced oxygen carrier is then transferred to the air reactor (AR), where is reoxidized with air. The combination of CLC with biomass as fuel can be considered a promising BECCS technology currently under development. Solid fuels commonly utilized in CLC encompass biomass^7^ and solid wastes, such as sewage sludge.^8^ Chemical looping with oxygen uncoupling (CLOU) is a method to enhance the kinetics of solid–solid reactions involving fuels and OCs. A recent review^9^ outlines the key properties of currently considered oxygen carriers, with a particular emphasis on the CLOU feature, which appears to be crucial for direct solid fuel combustion. The key idea behind CLOU is the utilization of certain oxygen carriers, such as CuO/Cu_2_O, Mn_2_O_3_/Mn_3_O_4_, and Co_3_O_4_/CoO, and their mixed oxides, which have the unique ability to release oxygen inside the FR.^10^ As a result, this released oxygen takes part in the combustion process and promotes efficient fuel oxidation. The reduced oxygen carriers are then transferred to the AR, where they undergo reoxidation through interaction with ambient air, allowing the now reoxidized oxygen carriers to be cycled back to the FR for reuse in subsequent cycles.^11^ Most of the studies regarding CLOU oxygen carriers are focused on copper-based oxides. Cu presents an economically viable price point, environmentally friendly attributes, the highest oxygen uncoupling capacity and notable reactivity, making it a good alternative to be considered in further research.^12−16^ The proof of concept for CLOU technology was demonstrated in a continuous 1.5 kW_th_ unit utilizing a copper oxide-based oxygen carrier.^17^ In that plant, full combustion of biomass was achieved using a copper oxide-based oxygen carrier at a temperature of 935 °C, along with a CO_2_ capture efficiency of 100%.^18^ Adánez-Rubio et al. developed a Cu–Mn mixed oxide oxygen carrier and assessed its performance in a CLOU unit. Remarkably, they achieved complete combustion of the fuel and attained a high CO_2_ capture efficiency of over 95% even at the relatively low temperature of 850 °C.^19^ In the same authors’ previous study, a Cu-based magnetic Fe–Mn-supported oxygen carrier (Cu30MnFe) developed by them was tested in a 1.5 kW_th_ CLOU unit. Besides achieving complete combustion in the FR, its magnetic properties enhanced its efficiency for separation from the ashes, with an achieved value of 98% reported.^20^ The magnetic permeability of the oxygen carrier decreased from 3.4 (−) to 3.2 (−) after 63 h of hot circulation in plant. The magnetic properties of oxygen carriers present an opportunity to enhance their separation from ashes, facilitating their reuse within the plant. In order to analyze the effect of doping with small amount of different inert oxides to improve the properties of Cu-based oxygen carriers, Abuelgasim et al.^21^ synthesized different Cu-based oxygen carriers by adding 5 wt % Na_2_O, K_2_O, CaO, and MgO, respectively, by means of sol–gel method testing in thermogravimetric analysis. They found that addition of Na_2_O and K_2_O had a negative effect on the uncoupling rate. In contrast, CaO and MgO addition increased the uncoupling rate. However, it is necessary to know their effect with regard to the mechanical behavior of the oxygen carrier. In addition, another study^22^ performed a screening procedure on magnetic CuO materials enhanced with kaolin to provide them with higher mechanical resistance for the purpose of the CLOU process, and a selected oxygen carrier was tested in a 1.5 kW_th_ CLOU unit. In terms of mechanical strength, kaolin addition increased crushing strength from 1.7 to 3.3 N, while the attrition rate of the kaolin-enhanced oxygen carrier retained almost constant throughout the campaign. Cu30MnFekao7.5 showed high reactivity, resulting in full combustion efficiency even at the low temperature of 800 °C with different biomasses without no problems of agglomeration. The oxygen carrier retained its magnetic properties after 80 h of hot circulation operation. Initially, the fresh oxygen carrier exhibited a magnetic permeability of 3.6. After extended combustion, this value decreased to 1.6, indicating that the Cu30MnFekao7.5 oxygen carrier remained sufficiently magnetic for separation from biomass ashes. As a result, 99.3% of the oxygen carrier particles could be successfully separated from the ashes, demonstrating its suitability for reuse in the plant.^23^

To make the chemical looping (CL) process and CLC with biomass viable for BECCS, more knowledge about the interactions between biomass ashes and oxygen carriers is needed, as it is well-known that biomass ash can be aggressive and corrosive during thermal conversion at high temperature. Interaction between ash and the oxygen carrier can decrease solid conversion and increase agglomeration in the bed material.^24^ Dai and Whitty^25^ studied the reaction between ash-derived Al_2_O_3_/Fe_2_O_3_ and copper oxide as the oxygen carrier during the combustion of coal. The effect of coal ash on agglomeration was found to be strongly related to ash composition, temperature and the ash to CuO ratio, and that IL6 coal ash deactivated around 15% of the copper oxide at 900 °C. The extent of sintering and deactivation increased by increasing the temperature. Several previous studies in the literature attempted to shed light on the complex interactions between oxygen carriers and ash constituents in biomass combustion processes. Darwish et al.^26^ examined the interaction between copper oxide-based oxygen carriers and ash components. They observed that a substantial portion of copper oxides remained intact without undergoing any interaction with the ash. However, the formation of silicate-based compounds, particularly potassium silicates, was found to contribute significantly to strong agglomeration phenomena. Another study by Staničić et al.^27^ considered the solid–state reaction of hematite, hausmannite, and synthesized ilmenite oxygen carriers with different ash components, such as calcium carbonate, silica, and potassium carbonate at 900 °C. The findings indicated that the oxygen carriers, specifically hausmannite and hematite, exhibited a more pronounced interaction compared to the synthesized ilmenite in terms of both physical attributes and discernible phases. Notably, synthesized ilmenite only formed new phases in systems incorporating potassium. Yilmaz and Leion^28^ investigated the interaction between iron-based oxygen carriers and alkaline salts found in biomass-derived ash. Their findings revealed that calcium deposits primarily formed on the outer layer of the iron-based oxygen carrier. Additionally, the presence of potassium and silicon resulted in the formation of bridges between ash components and the oxygen carrier, leading to increased agglomeration. The effect of potassium salt on ilmenite was investigated. In redox experiments conducted at 850 °C, Hildor et al.^29^ found that potassium salts, such as K_2_CO_3_, K_2_SO_4_, and KCl, increased the reactivity of ilmenite, and that KPO_3_ formed a layer around the oxygen carrier, leading to agglomeration. This KPO_3_ layer is almost impermeable to CO and decreases reactivity toward H_2_. Another work^30^ examined the interaction between two oxygen carriers diluted with silica sand, one LD slag and the other ilmenite, and potassium salts in a fixed bed at 900 °C. The interaction of the potassium salt with the oxygen carrier resulted in agglomeration in the setup. The interaction between iron oxygen carriers and biomass ash-forming compounds was studied.^31^ The results showed that during reduction and oxidation reactions at 950 °C, calcium and magnesium nitrate and phosphate caused the agglomeration of iron-based oxygen carriers. Mei et al.^32^ investigated the effect of the alkali components of potassium and the sodium from biomass on a braunite manganese ore oxygen carrier in a batch fluidized bed reactor. Partial agglomeration and defluidization were expected and found to occur on the surface of particle exactly where, based on EDX results, the alkali components of the sodium and potassium were located. In fact, the accumulation of potassium can lead to soft agglomeration within the combustion bed.^33^ It was observed that the ash elements in biomass, particularly components with low melting points, such as sodium and potassium, as well as compounds with high melting points, such as calcium and magnesium, exhibit inhibitory properties on oxygen carriers.^34^ In another study conducted by Liu et al.,^35^ it was discovered that potassium can induce agglomeration within the bed. Nevertheless, it was also observed that potassium has a positive impact on expediting the conversion of biomass. Purnomo et al.^36^ studied the interaction between potassium salts, representative of the most important biomass ash compounds, and an iron-based oxygen carrier. The experiments involved mixing the oxygen carrier material with a potassium salt (K_2_CO_3_, KH_2_PO_4_, and K_2_SO_4_, respectively) in an alumina crucible at high temperature under reducing conditions. The potassium content in the mixture (4 wt %) approached the lower detection limit of the diffractometer, posing challenges for definitively identifying any potassium phases in the samples via X-ray diffraction (XRD). According to the scanning electron microscopy with energy dispersive X-ray analysis (SEM–EDX) results, the experiments involving iron sand showed that potassium from all three different potassium salts consistently diffused into the iron sand particles. Among the tested materials, it is evident that iron sand consistently exhibited some degree of agglomeration after exposure to any potassium salt. The high silica content in iron sand likely contributes to its agglomeration, as silicon and potassium have a strong affinity for each other, forming potassium silicate, which has a low melting point.

Andersson et al.^37^ considered gaseous alkali interactions with ilmenite, manganese oxide, and calcium manganite in the fluidized bed at 900 °C. The type of oxygen carrier plays a crucial role in alkali uptake, with ilmenite showing almost complete uptake of alkali, particularly under reduction conditions. Overall, the experimental results showed the complex behavior of alkali and alkaline materials in CLC. Therefore, it is crucial to consider the interaction between fuel ash and CLOU oxygen carriers. This work studied the interaction of two magnetic copper-based oxygen carriers, Cu30MnFe and Cu30MnFekao7.5, with pine sawdust biomass during combustion in a 1.5 kW_th_ continuous unit under CLOU conditions.

## 2 Experimental Section

### 2.1 Oxygen Carrier Preparation

Two distinct oxygen carriers were synthesized using a spouted fluidized bed spray granulator (Glatt W51530 + OPP1). Raw components were used, such as CuO (Panreac), Mn_3_O_4_ (Micromax, Elkem), Fe_2_O_3_ (Acros Organics), and kaolin (Sumitomo Seika), along with different additives including poly(ethylene oxide) (PEO-1, Sumitomo), dispersing agents and deflocculants. Both oxygen carriers were produced with the same amount of CuO as the active phase (30 wt % CuO). In addition to CuO, the first comprised 30.55 wt % Mn_3_O_4_, 31.95 wt % Fe_2_O_3_, and 7.5 wt % kaolin, whereas the second contained 34 wt % Mn_3_O_4_ and 36 wt % Fe_2_O_3_. The oxygen carriers are denoted as Cu30MnFekao7.5 and Cu30MnFe, respectively. The Cu30MnFekao7.5 granules were subjected to calcination at 1050 °C for 4 h, while the Cu30MnFe was calcined at 1100 °C for 4 h. The two calcined oxygen carriers were sieved to obtain particles within the desired range of 100–300 μm.

### 2.2 Oxygen Carrier and Pine Sawdust Characterization and Data Evaluation

Key properties of the synthesized oxygen carriers are presented in Table 1. The magnetic permeability (μ) listed in the table was calculated based on the magnetic volumetric susceptibility value χ_v_ (m^3^/kg), which was measured using a Bartington single frequency MS2G sensor connected to a magnetic susceptibility MS3 meter.
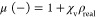
1

**Table 1:** Properties of Fresh and Used Cu30MnFekao7.5 and Cu30MnFe

	Cu30MnFe		Cu30MnFekao7.5	
	fresh	used	fresh	used
CuO content (wt %)	30	30	30	30
oxygen transport capacity, *R*_OC_ (wt %)	2.4	2.8	2.3	2.4
crushing strength (N)	1.49	1.12	2.3	1.43
magnetic permeability μ (−)	4.7	2.4	3.6	2.3
skeletal density (kg/m^3^)	5125	5070	4720	4617
porosity (%)	6.9	26.39	29.5	26.10
air jet attrition index (AJI) (%)	0.4	2.8	0.2	3.4

A Shimpo FGN-5 digital force gauge apparatus was employed to determine the crushing strength of the oxygen carriers, measured as the force necessary to fracture a particle. The reported crushing strength values in this study represent the average of a minimum of 20 measurements conducted on randomly selected particles falling within the specified range of 100–300 μm.

In this study, XRD was employed to determine the crystalline solid phases present in our designated samples. The corresponding spectra were captured using a Bruker D8 Advance X-ray powder diffractometer, outfitted with a Cu anode X-ray source operating at 40 kV and 40 mA, alongside an energy-dispersive one-dimensional detector. The diffraction pattern was obtained within the 2θ range of 10–80°, with a step size of 0.019°. To identify the phases within the oxygen carriers, we utilized DIFFRAC.EVA software, augmented by a comprehensive reference pattern database sourced from the Crystallography Open Database (COD) and the Powder Diffraction File (PDF). This approach facilitated precise phase identification, enabling a thorough analysis of our samples.

Inductively coupled plasma optical emission spectrometry (ICP-OES) was performed by means of an Xpectroblue-EOP-TI FMT26 (Spectro) to determine the elements in the ash and prepared oxygen carriers in a range of quantifiable concentrations.

Selected samples were observed under a field emission scanning electron microscope (SEM, Carl Zeiss MERLIN with a hot cathode field emission electron gun) and equipped with an energy-dispersive X-ray spectroscopy (EDX) detector for analysis of the energy of scattered X-rays X-max (20 mm^2^) with silicon drift detector from Oxford instruments. The samples were embedded in an epoxy mold, after which the epoxy surface was meticulously polished to produce the cross sections of the particles.

A three-hole ATTRI-AS air jet attrition resistance tester (MaTec Materials Technologies Snc) was utilized to evaluate the attrition resistance of each oxygen carrier in terms of its attrition jet index (AJI) as per the American standard test method D5757-22.^38^ In accordance with this protocol, weight losses (fines) from 50 g of each oxygen carrier were recorded under an airflow of 10 L/min after 5 h of operation. This facilitated the calculation of AJI as the percentage of fines after 5 h, where AJI is calculated using the following formula
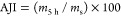
2where *m*_5 h_ represents the mass of fines sized less than 40 μm collected after 5 h, and *m*_s_ represents the mass of the sample loaded into the apparatus.

Helium pycnometry is the method used to determine the skeletal density of solid materials by measuring the amount of helium displaced by the sample, using a micromeritics ACCUPYC II instrument. Different working volumes, are available, allowing for the analysis of varying sample quantities. The temperature of the analysis chamber can be controlled, enabling density determination at different temperatures which was 30 °C.

In the specific surface area test conducted on both oxygen carriers using the Brunauer–Emmett–Teller method, both fresh and in postexperiment conditions, the measured values were found to be below 0.5 m^2^/g.

*R*_OC_ refers to the maximum amount of oxygen that an oxygen carrier can transport during a CL process. In this study, copper oxide and its mixed oxides were assumed to be the active phase responsible for oxygen uncoupling. It is important to note that under the specified conditions,^39^ the oxidation of MnFe mixed oxide to the bixbyite phase, which also possesses oxygen uncoupling capability, is hindered. Therefore, any oxygen uncoupling capability observed in the prepared materials was assumed to come from the CuO or a mixed oxide containing copper, manganese and iron.

Pine sawdust (*Pinus sylvestris*), milled and sieved 0.5–2 mm, was used as fuel biomass during combustion. Table 2 shows the properties of the pine sawdust after analysis.

**Table 2:** Main Properties of Pine Saw Dust

	(wt %)		(wt %)
humidity	6.9	C	47.5
volatiles	73.6	H^a^	5.6
fixed carbon	17.9	N	0.3
ash	1.6	S	<0.11
LHV (kcal/kg)	4384		

a H free of moisture.

Combustion efficiency was calculated based on the amount of oxygen required to consume unburnt gases from the FR, and the oxygen required for complete combustion of solid fuel in the FR, as follows
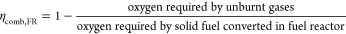
3

CO_2_ capture efficiency, η_cc_, was defined as the ratio of CO_2_ exiting the FR and the carbon exiting as CO_2_ at the FR and AR outlets.
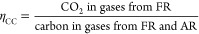
4

### 2.3 Experimental Setup and Experimental Conditions

The 1.5 kW_th_ CLOU unit consisted of two interconnected bubbling fluidized beds, serving as FR and AR, along with a loop seal designed to prevent the mixing of gases between these reactors. It also featured a cyclone and a solid control valve. An air–N_2_ mixture was utilized at a rate of 2100 LN/h inside the AR, while N_2_ was used inside the FR and loop seal at rates of 180 LN/h and 100 LN/h, respectively. A detailed description of the plant can be found in previous research works.^17^

The aim of the present experimental campaign was to analyze the possible interaction between biomass ashes and the oxygen carriers used. In this respect, the experimental conditions were configured to allow the corresponding experiments to achieve high combustion efficiency and CO_2_ capture efficiency, and then to remain unchanged throughout the whole experimental campaign. The experimental setup maintained a constant fuel feeding rate of 120 g/h throughout the experiments. Simultaneously, the oxygen carrier circulation rate remained steady at 25 kg/h. The temperature within the system was regulated and kept constant at 900 °C in both the FR and AR throughout the experiments.

Furthermore, the inventory of oxygen carrier material present in the plant was 4 kg. In order to analyze possible interactions ash-oxygen carrier, experiments should be run over a long period. In the case of Cu30MnFekao7.5, the experiments involved 56 h of combustion operation with pine sawdust as fuel and 105 h of hot fluidization. The experiment using Cu30MnFe comprised 16 h of combustion and 35 h of hot circulation.

## 3 Results and Discussion

### 3.1 Experimental Parameters

In all the pine sawdust combustion experiments performed in the 1.5 kW_th_ CLOU continuous unit, a combustion efficiency of 100% was achieved with both of the oxygen carriers tested in this work, confirming their high reactivity.

**Figure 1:**
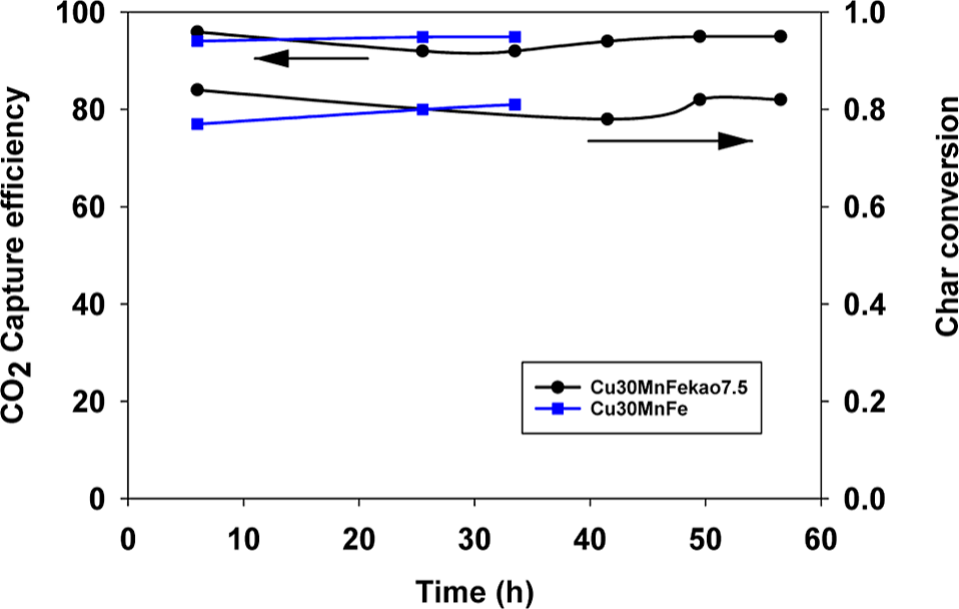
CO_2_ capture percentage and char conversion of both oxygen carriers Cu30MnFekao7.5 and Cu30MnFe during combustion operation time.

Figure 1 illustrates the CO_2_ capture percentage during the hours of combustion for Cu30MnFekao7.5 and Cu30MnFe. It is evident that in both cases, the CO_2_ capture percentage remained nearly constant at a high value of approximately 95%.

Furthermore, Figure 1 depicts the char conversion achieved by the two oxygen carriers. Similarly, to the stable CO_2_ capture percentage observed during combustion operation, char conversion for both oxygen carriers remained steady at approximately 0.8. The values shown in Figure 1 are in line with those previously obtained in pine sawdust combustion with the same oxygen carriers in previous works by the authors.^20,23^

### 3.2 Analysis of Ash-Oxygen Carrier Interaction

The pine sawdust ash components were first determined using ICP-OES, whose results are shown in Table 3. The biomass ash analyzed was obtained from the proximate analysis and then digested with Li_2_B_4_O_7_ in order to analyze by ICP-OES.

**Table 3:** Pine Sawdust Ash Component^a^

pine sawdust ash component	wt %	pine sawdust ash component	wt %
Ca	22.8	Al	1.34
K	7	P	0.67
Si	6.33	Na	0.21
Mg	2.61	Mn	0.15
Fe	1.56	Ti	0.09

a O is the element required to complete the equilibrium.

The main ash components in pine sawdust ash were Ca, K, Si, and Mg. The methodology used to analyze possible interactions between these ash components and the oxygen carriers was the extraction of used oxygen carrier samples after different combustion times and their subsequent characterization by ICP-OES to determine their composition and SEM–EDX to analyze their changes in morphology and/or distribution of their constituent elements. In order to analyze possible interaction between oxygen carrier with ashes, oxygen carrier particles were separated from independent ash particles in the bed by magnetic separation, prior to analysis with ICP-OES and SEM–EDX, as can be seen in Figure S1, and only oxygen carrier particles can be found in the analyzed sample. The ICP-OES composition obtained for the used oxygen carriers was expected to provide a first indication of any interaction or presence in case any of these elements significantly increased their presence in the used oxygen carrier particles. Table 4 shows the ICP-OES results for both fresh and used oxygen carriers, which focus on the main elements identified in the biomass ashes according to, i.e. Ca, K, Si, and Mg. In all cases P was found to be below the ICP-OES quantitative level (<0.04%). Differences in the composition of the fresh oxygen carriers can be observed in Table 4 regarding the amounts found for elements K, Si, and Mg. This is due to the fact that these elements are components of the kaolin used in the preparation of the Cu30MnFekao7.5 material. The ICP-OES analysis performed on the kaolin sample indicated 2.68 and 19.13 wt % for K and Si, respectively.

**Table 4:** ICP Results of Fresh and Used Cu30MnFe and CuMnFekao7.5 Oxygen Carriers (wt %)

samples	Ca	K	Si	Mg
Cu30MnFe-fresh	0.12 ± 0.04	<0.04	0.4 ± 0.03	0.06 ± 0.07
Cu30MnFe-used_15 h	<0.05	<0.04	0.48 ± 0.03	0.05 ± 0.07
Cu30MnFekao7.5-fresh	0.12 ± 0.04	0.12 ± 0.01	2.53 ± 0.03	0.18 ± 0.07
Cu30MnFekao7.5-used_15 h	0.12 ± 0.04	0.15 ± 0.01	2.50 ± 0.03	0.25 ± 0.07
Cu30MnFekao7.5-used-56 h	0.10 ± 0.04	0.28 ± 0.01	2.7 ± 0.03	0.38 ± 0.07

**Figure 2:**
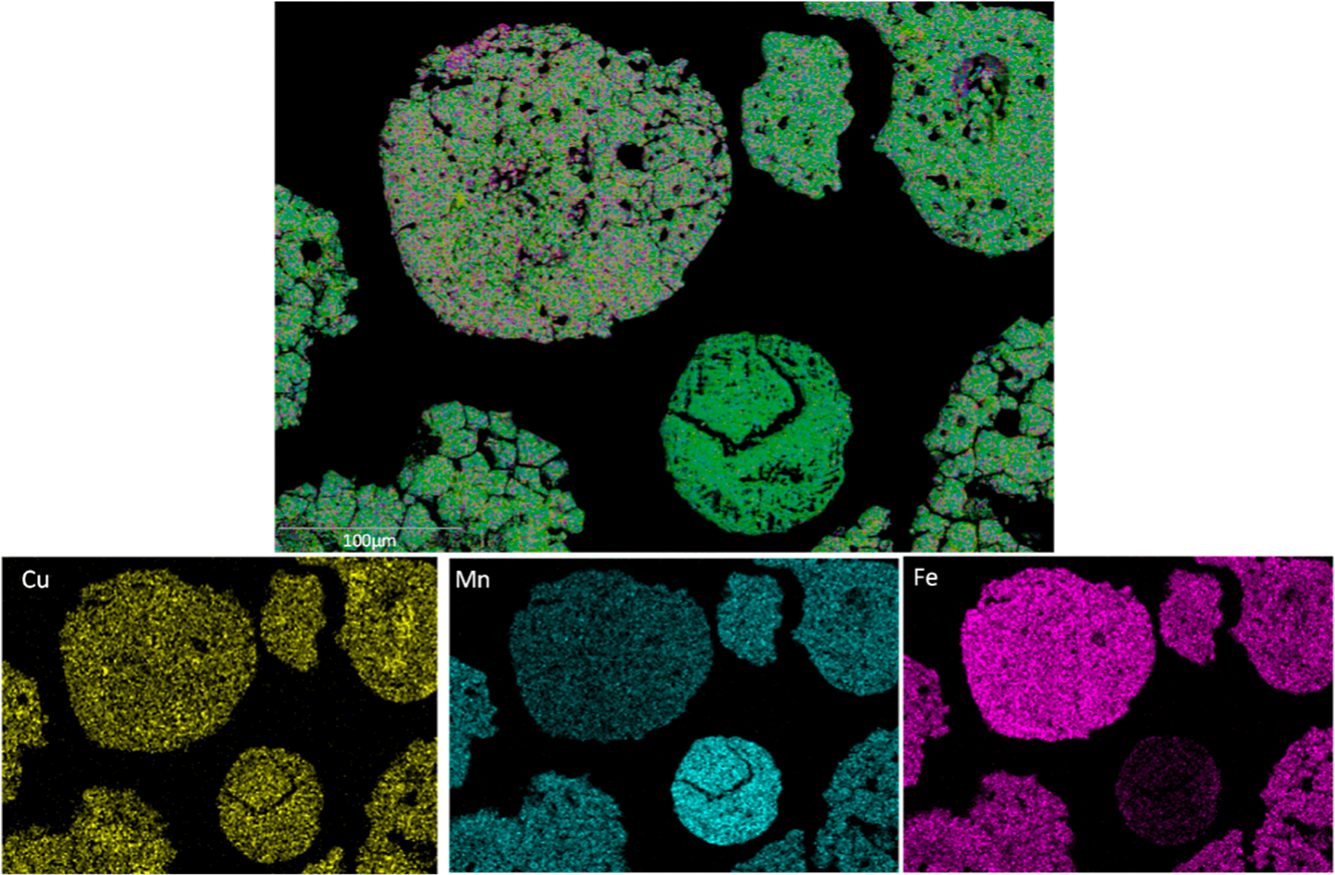
SEM–EDX mapping of used Cu30MnFe.

**Figure 3:**
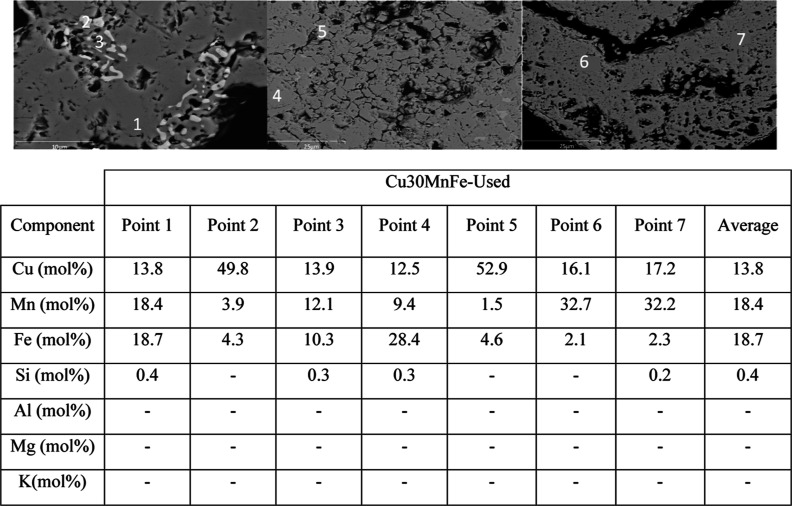
SEM–EDX backscattered electron (BSE) images of a cross-section of a used Cu30MnFe particles including composition at different points.

In the case of the Cu30MnFe oxygen carrier, no significant increase was observed in the amount of Ca, Si, Mg, and K after 15 h of pine sawdust combustion, see Table 4, and therefore no sign of interaction could be assumed. This fact, together with the mapping result of the used Cu30MnFe, Figure 2, and different elemental points composition on SEM–EDX image conducted, Figure 3, it can be concluded that no interaction was found to take place between the oxygen carrier and alkaline metal.

As shown in Figure 3 and in the accompanying table, which presents data from various points on different particles, no ash elements, such as Mg or K, were detected on different parts of the Cu30MnFe particles used. This observation corroborates the quantitative ICP-OES results, confirming that the total amount of ash elements in the oxygen carrier was almost negligible and that there was no interaction between the Cu30MnFe oxygen carrier and ash components. Furthermore, Figures S2 and S3 show SEM–EDX mapping of the fresh and used Cu30MnFe, respectively, to compare element distribution in both cases.

Because of the unstable operation with Cu30MnFe observed in the continuous unit and attributed to the high generation of fines, see Figure S1b, the experimental campaign was only able to continue with the Cu30MnFekao7.5 oxygen carrier. However, in the case of the Cu30MnFekao7.5 and after the same time of pine sawdust combustion, the results shown in Table 4 could indicate the accumulative presence of biomass ashes, given that there is an increase in the amount of K and Mg in the oxygen carrier. After 56 h of pine sawdust combustion with this material, no significant changes were observed in the amount of Ca found in the used oxygen carrier, which is the main element present in the ashes. Therefore, the absence of interaction between the oxygen carrier and Ca, as the main component of ash, indicate that the greater part of the ashes can be separated without problems owing to the magnetic properties of the Cu30MnFekao7.5, as was previously observed by Adánez-Rubio et al., who obtained a magnetic separation efficiency of the oxygen carrier from ash of 99.3%.^23^ On the other hand, an increase was observed in the presence of Mg from 0.18 to 0.38 wt %. The amount of K also increased from 0.12 to 0.28 wt %. These results could indicate that an accumulative presence of pine sawdust ashes was taking place in the Cu30MnFekao7.5 oxygen carrier. Consequently SEM–EDX analysis was performed to look for further evidence of this presence.

**Figure 4:**
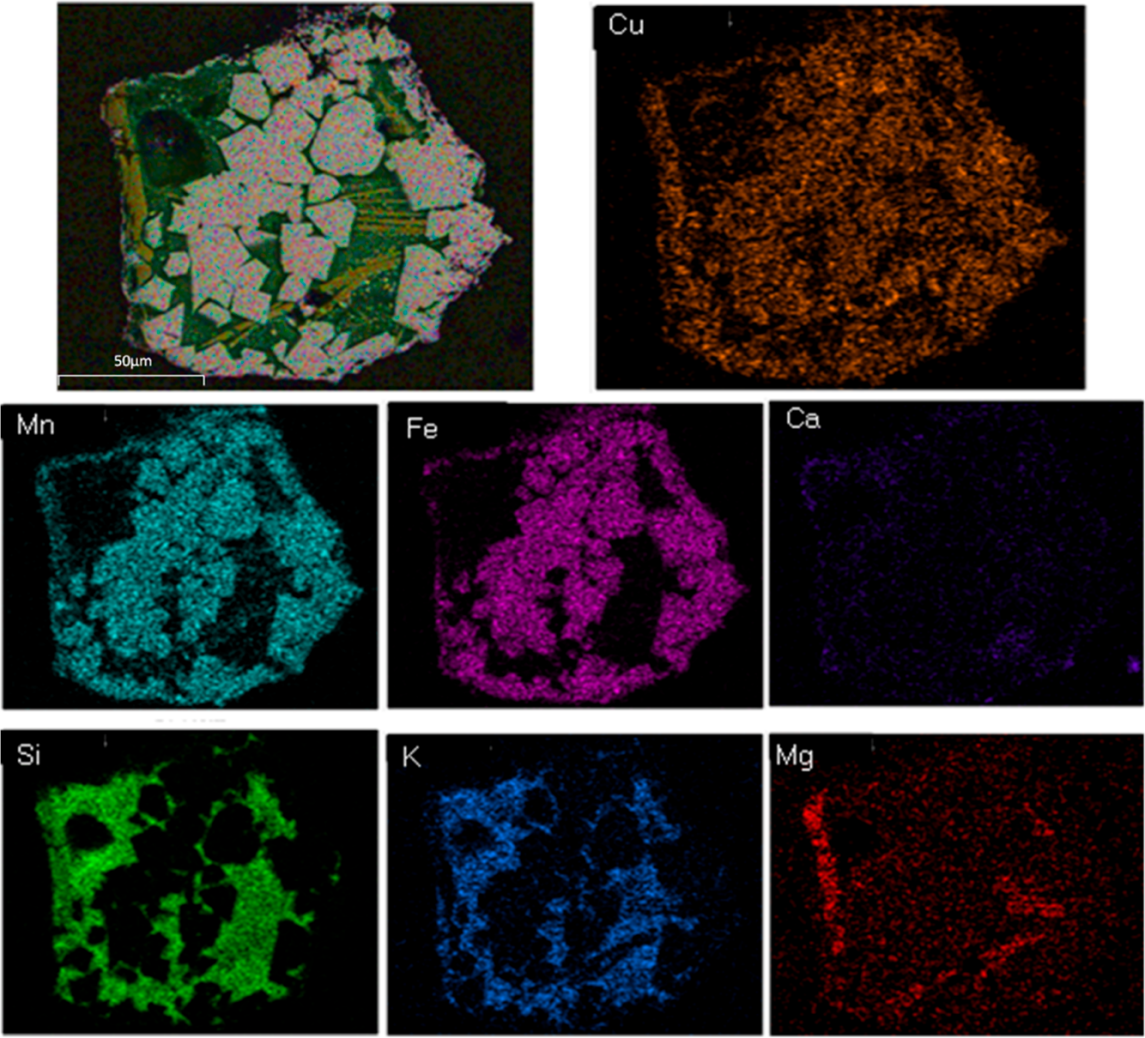
SEM–EDX elemental mapping of used Cu30MnFekao7.5 oxygen carrier after 56 h of pine sawdust combustion.

Figure 4 shows the distribution of different elements on one used Cu30MnFekao7.5 oxygen carrier particle after 56 h of pine sawdust combustion. Different areas can be observed on the particle, some of which are enriched with Cu–Mn–Fe and others with kaolin. With regard to the latter, the fact that kaolin already contains Si, Al, K, and Mg makes it difficult to observe any changes attributed to the interaction between the particles and biomass ashes. Accumulations of these elements on the particle surface could only be inferred in the case of Mg. In addition, it seems that K can penetrate into the oxygen carrier particles, diffusing in the kaolin-rich areas, without interaction with the active phase. The presence of K in regions devoid of copper oxide, manganese oxide, and iron oxide, yet containing Si, is illustrated in Figure 4. Additionally, a small amount of Mg can be detected, mainly on the surface of the particle.

**Figure 5:**
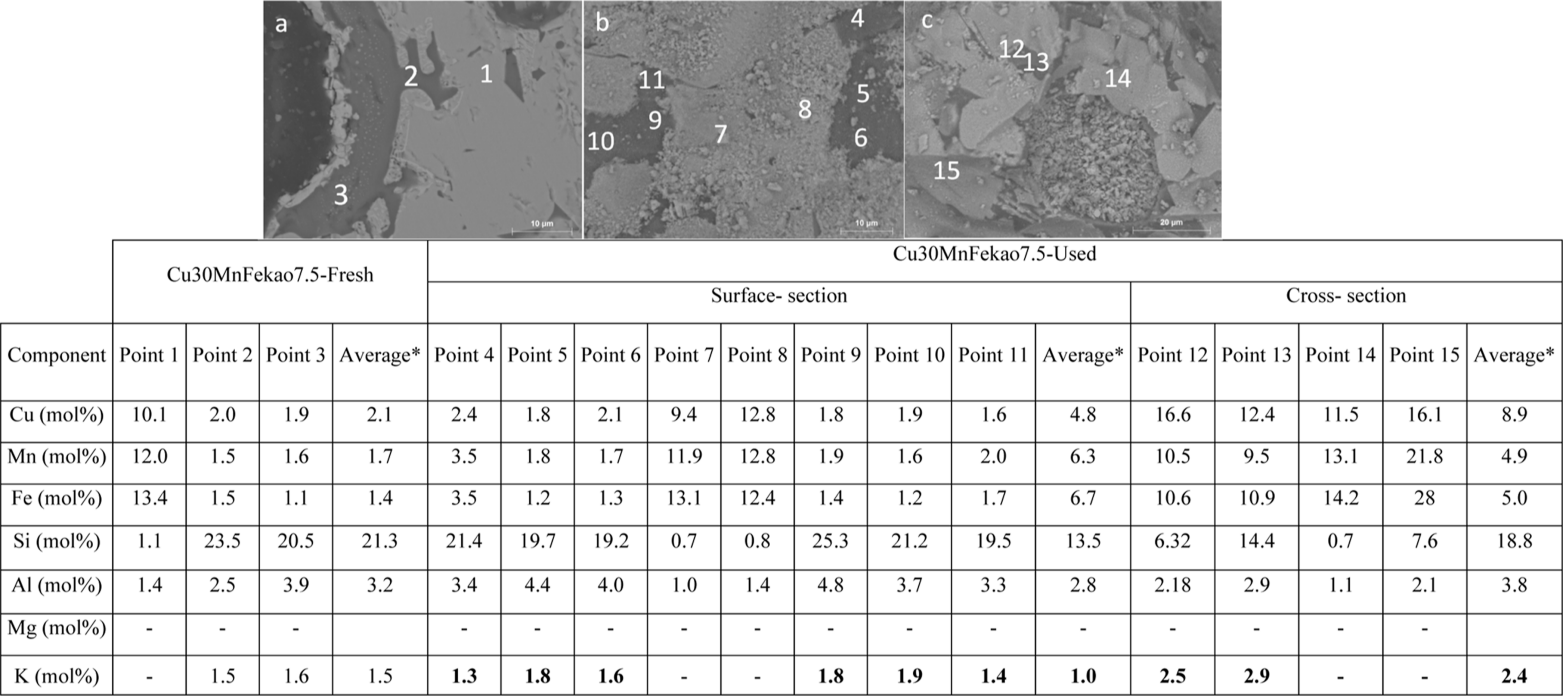
SEM–EDX BSE images of (a) cross-section of fresh Cu30MnFekao7.5 and (b) surface-section of used Cu30MnFekao7.5, and (c) cross-section of used Cu30MnFekao7.5 after 56 h of pine sawdust combustion, including table of main component changes at different points. * The average value for each component is calculated based on the concentrations of the component at the points in the fresh and used oxygen carrier, as indicated in Figures 5b and S6.

**Figure 6:**
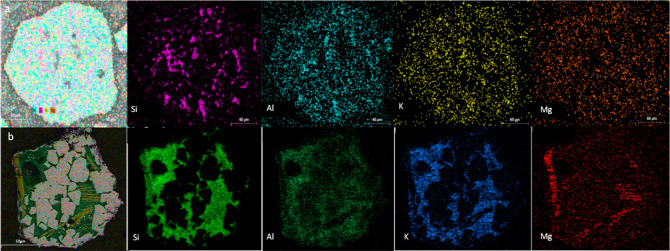
Result of SEM–EDX elemental mapping of the main elements found in the ashes after pine sawdust combustion: (a) fresh Cu30MnFekao7.5, (b) used Cu30MnFekao7.5.

Furthermore, Figure 5a shows BSE images of fresh Cu30MnFekao7.5 oxygen carrier, Figure 5b,c shows the same oxygen carrier after 56 h of combustion. According to the EDX results, accumulative K could be observed in the dark areas of Figure 5, where there was a high kaolin concentration. As observed at points 12, and 13, where Si and Al are present inside the used oxygen carrier Cu30MnFekao7.5, K also appears. Conversely, at point 14, where the concentration of Si and Al, indicative of kaolin, is low, there is no trace of K. Figure S6 presents additional points in the used particles. As can be seen in Figures 5b and in S6a,b the presence of K in the surface is similar or even lower (due to the surface was enriched in Mn, Fe, and Cu, see Figure 6a,b) than the found in the inner of the particle. The average value of 2.4 mol % for K inside the used particles is calculated based on the data from the used oxygen carrier shown in Figures 5 and S6. This result demonstrates an accumulation of K related to the kaolin in the used particles compared to the fresh oxygen carrier, which has an average value of 1.5 mol %.

Figure 6a,b shows the elemental mapping of fresh and used Cu30MnFekao7.5 with regard to the main elements found in the ashes. In the fresh oxygen carrier, the presence of ash components, such as Mg, Ca, and K, is diffuse, and they are detected over the entire particle; only a small higher presence of these can be seen in areas enriched with Si and Al. Conversely, the SEM–EDX results in Figure 6b for the used oxygen carrier reveal that K is predominantly found in regions associated with kaolin, even in the center of the particle, involving the elements Al and Si. It can therefore be said that some kind of interaction has taken place, although given the small change in composition, it is not possible to indicate whether this was due to chemical interaction or simply diffusion of K owing to greater solubility in the areas of kaolin inside the particles. Moreover, Mg is mainly found to be more present on the surface of the particle together with only Si.

**Figure 7:**
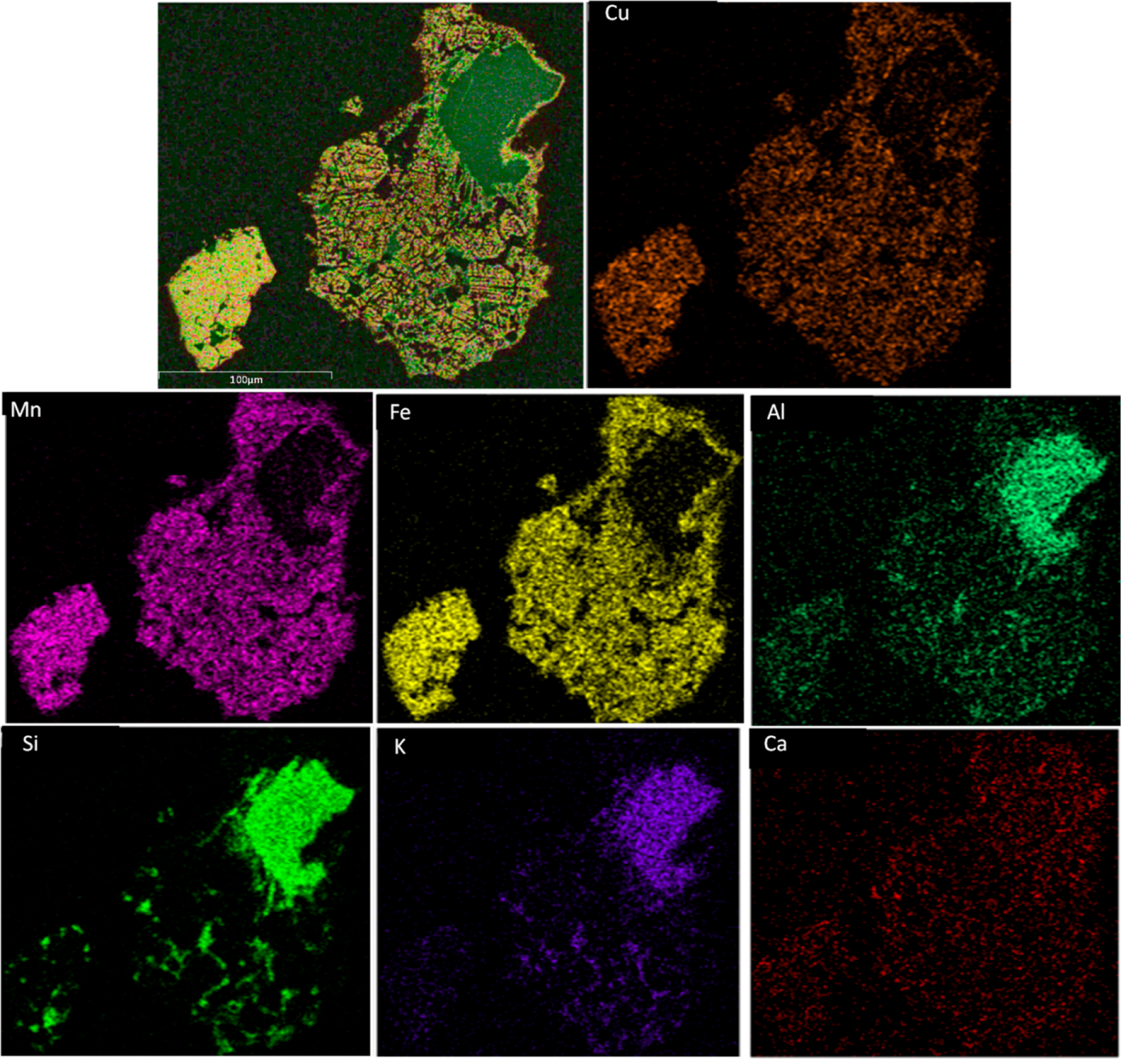
Result of SEM–EDX elemental mapping of used Cu30MnFekao7.5 after pine sawdust combustion.

Figure 7 shows another SEM–EDX elemental mapping of used Cu30MnFekao7.5 and indicates the presence of K in regions containing kaolin. Similar K penetration into the oxygen carrier, as shown in the experimental results, was previously observed by Purnomo et al.^36^ in their experiments using iron sand, an oxygen carrier enriched with silica (16 wt %) and potassium salts (K_2_CO_3_, KH_2_PO_4_, and K_2_SO_4_), they found that K diffused into the individual particles of the oxygen carrier. The iron sand exhibited agglomeration after exposure to any potassium salt, which can be attributed to the interaction of K with its high silica content. In the present study, although K was present within the particles, agglomeration did not occur during the pine sawdust combustion. Figures S4 and S5 present SEM–EDX elemental mappings of additional particles of fresh and used Cu30MnFekao7.5, respectively.

**Figure 8:**
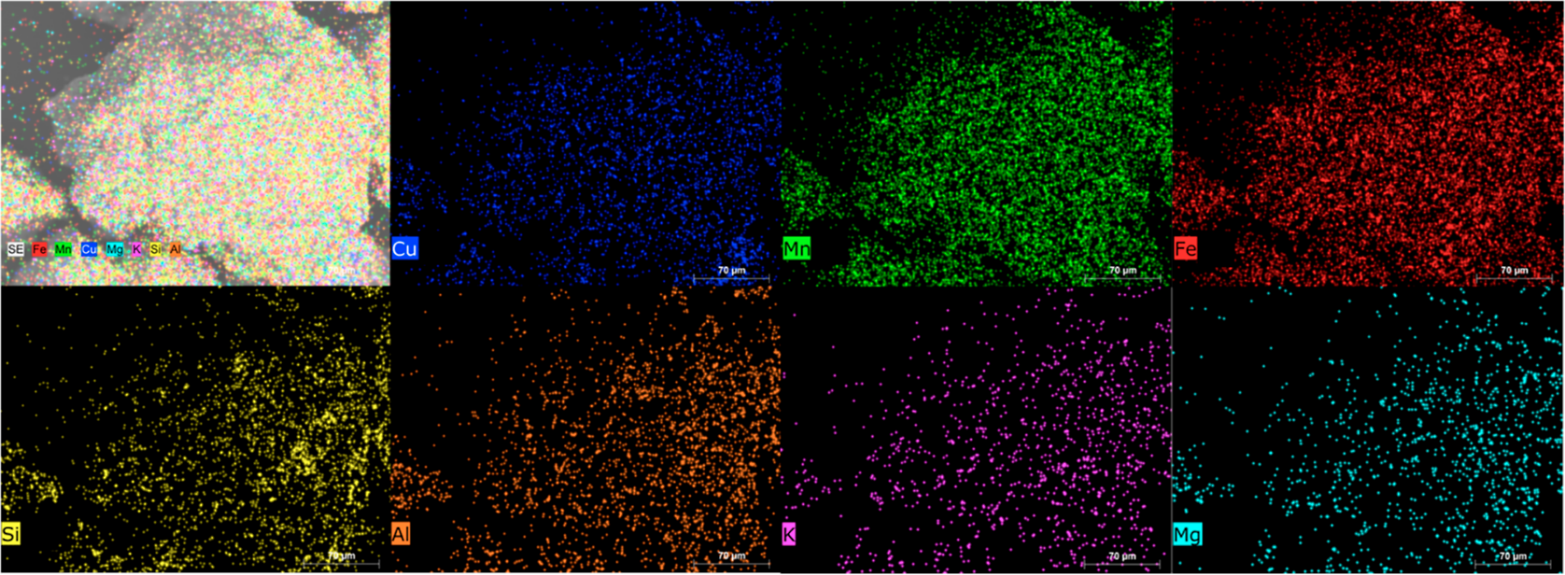
SEM–EDX elemental mapping image of the surface of used Cu30MnFekao7.5 after pine sawdust combustion.

In order to analyze the presence of K in the surface of the particle, Figure 8 illustrates the SEM–EDX elemental mapping of the surface of the used oxygen carrier, Cu30MnFekao7.5. The analysis reveals the presence of small amounts of Al and Si on the particle surface, while no noticeable accumulation of K is observed. Similarly, Figure S8 presents the SEM–EDX elemental mapping of another particle of the same oxygen carrier, exhibiting an elemental distribution pattern consistent with that shown in Figure 8. As it can be seen the surface is enriched in Mn, Fe, and Cu, observing less proportion of kaolin in the surface and therefore low amount of K in the surface. So, it can be concluded that there was not a surface accumulation of K due to the low presence of kaolin in it.

**Figure 9:**
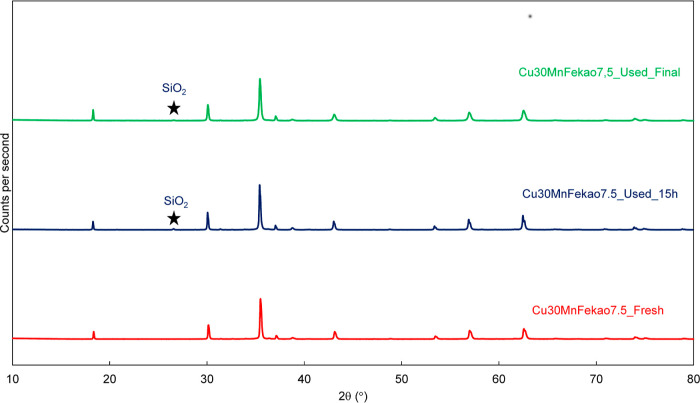
XRD patterns for fresh Cu30MnFekao7.5 and used after 15 and 56 h of pine biomass combustion.

To further elucidate the implications that the interaction between biomass ashes and the used Cu30MnFekao7.5 could have, XRD tests were performed for fresh and used oxygen carrier samples (after 15 h and after 56 h of combustion with pine sawdust). The XRD patterns shown in Figure 9 indicate that the fresh and used oxygen carriers exhibit similar phases and that only free SiO_2_ appears in the Cu30MnFekao7.5 after several hours of operation. Thus, the possibility of new crystalline phases formation owing to interaction between the oxygen carrier and Mg or K cannot be confirmed by the analysis performed. Cu does not seem to interact with the ash constituents.

Previous studies indicated that the accumulation of K can lead to soft agglomeration within the combustion bed,^33^ with the presence of K and Si resulting in the formation of bridges between ash components and the oxygen carrier, leading to increased agglomeration. In a study on the interaction between copper oxide-based oxygen carriers and ash components,^26^ the formation of potassium silicates was found to be the cause of agglomeration. However, it should be highlighted that during the 56 h of pine combustion with Cu30MnFekao7.5, no signs of defluidization or agglomeration problems were found.

### 3.3 Evolution of Other Oxygen Carrier Properties

It should be mentioned that the magnetic properties attributed to the oxygen carriers can in fact be attributed to the presence of the spinel phase.^40^ These magnetic properties aid the effective separation of ash from particles and the recovery of the used oxygen carrier. According to Table 1, it is noteworthy that although the magnetic properties of the two oxygen carriers decreased over the course of the experiments, both oxygen carriers still exhibited sufficient magnetic properties by the end of the operation. This characteristic enabled the effective separation of the oxygen carriers from the ashes, facilitating their recovery and potential reuse in subsequent cycles.^20,23^

**Figure 10:**
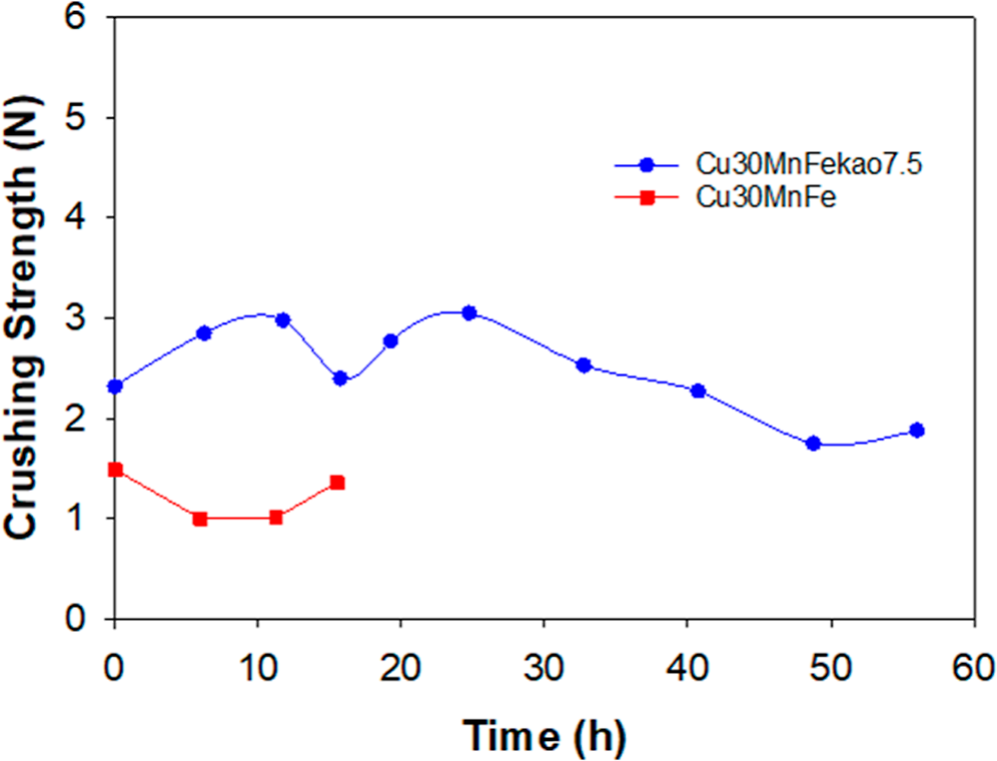
Evolution of mechanical strength of Cu30MnFe and Cu30MnFekao7.5 during operation.

The mechanical strength of oxygen carriers after operation hours was evaluated, as shown in Figure 10.

The mechanical strength of both oxygen carriers decreased during operation, as indicated by the results in Figure 10. The decrease observed in the case of Cu30MnFe can be attributed to an increase in porosity; however, for Cu30MnFekao7.5, this decrease cannot be directly linked to the evolution of porosity over time, as shown in Table 1. Nevertheless, by the end of the experiments, mechanical strength values were above values of 1 N, which is considered acceptable for operation.^41^ As can be observed, the addition of kaolin improved the mechanical strength of the oxygen carrier.^22^ An increase in oxygen transport capacity was observed after the combustion process for both oxygen carriers. In the case of Cu30MnFekao7.5, this increase had already been indicated in previous study.^23^ Furthermore, XRD results demonstrate free copper oxide in Cu30MnFe after combustion operation, which confirms the increase in oxygen transport capacity, see Figure S9.

## 4 Conclusion

Two oxygen carrier were prepared with a spouted fluidized bed spray granulator and the calcined materials were tested in a 1.5 kW_th_ CLOU continuous unit at 900 °C with pine sawdust under similar experimental conditions. The combustion parameters and interaction between both oxygen carriers and the biomass ash component of pine sawdust were investigated. Full combustion was achieved for both Cu30MnFe and Cu30MnFekao7.5 oxygen carriers, while CO_2_ capture efficiency was around 95% in the 1.5 kW_th_ continuous CLOU unit. By the end of the combustion process both oxygen carriers had retained their magnetic properties, which facilitated separation of the oxygen carriers from the ash components. The interaction between ash components and oxygen carrier was analyzed by ICP-OES and SEM–EDX analyses. ICP-OES analysis determined that the Cu30MnFe did not interact with biomass ashes after 15 h of combustion. However, the amount of Mg and K detected in the Cu30MnFekao7.5 particles doubled after 56 h of combustion. In addition, K seemed to be able to penetrate into the oxygen carrier particles, diffusing in the rich kaolin areas, without interaction with the active phase. It can therefore be said that some kind of interaction took place, although given the small change in composition found, it is not possible to indicate whether there was chemical interaction or simply diffusion of K due to its greater solubility in the kaolin areas inside the particles. Cu did not seem to interact with ash constituents. The oxygen carriers largely maintained their reactivity after the experiments. No agglomeration or fluidization problem were observed throughout the entire experimental campaign, even despite the accumulation of K in the oxygen carrier.

## Supporting Information Available

SEM results of fresh and used Cu30MnFe and Cu30MnFekao7.5 oxygen carriers (PDF)
