# Supplementary material for: Ash Interaction with Two Cu-Based Magnetic Oxygen Carriers during Biomass Combustion by the Chemical Looping with Oxygen Uncoupling Process
Source: Energy Fuels. 2024 Oct 4;38(20):19548–58. doi: 10.1021/acs.energyfuels.4c02464 (PMC11492256; doi:10.1021/acs.energyfuels.4c02464)
Supplement: Supplementary file 1 — ef4c02464_si_001.pdf [file ef4c02464_si_001.pdf]

Supplementary Material

Ash interaction with two Cu-based magnetic oxygen carriers during biomass combustion by the CLOU process

A. Filsouf, I. Adánez-Rubio*, T. Mendiara, A. Abad, J. Adánez

Carboquimica Institute, Miguel Luesma Castán, 4, 50018, Zaragoza, Spain

*Email address: iadanez@icb.csic.es

After 15 h of pine sawdust combustion, in the case of the Cu30MnFe oxygen carrier shows unstable operation in the continuous unit and attributed to the high generation of fines as it can be seen in Figure S1b, where fines particles can be seen together with oxygen carrier particles partially broken. However, it was not detected physical deposition of ashes over the particle surface in both cases of used oxygen carriers Cu30MnFe and Cu30MnFekao7.5, see Figures S1b and S1d, respectively.

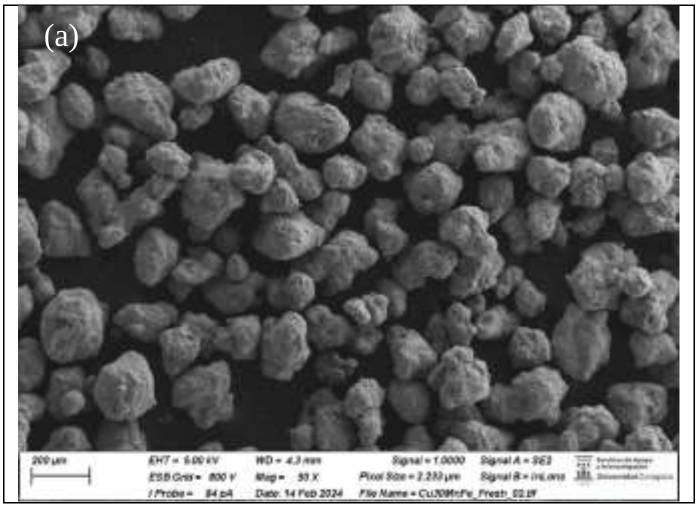

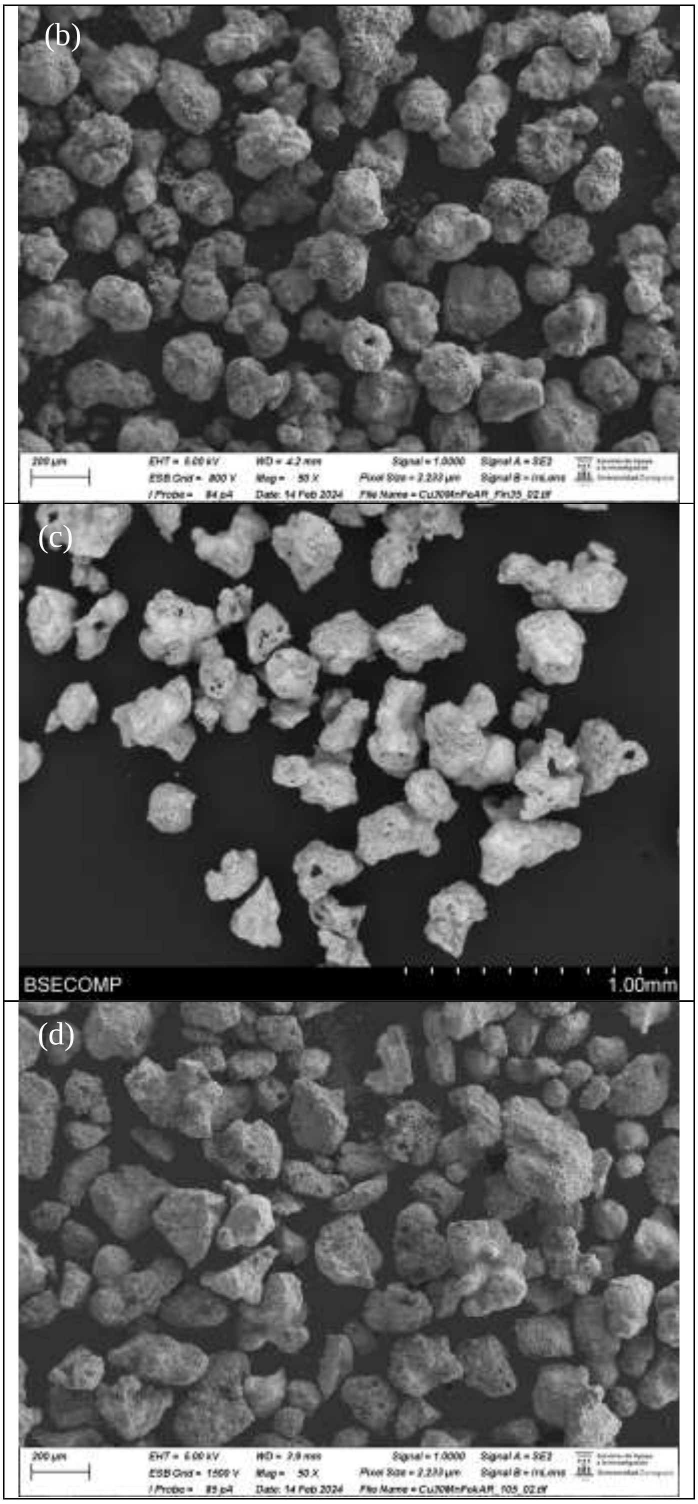

Figure S1. SEM-EDX SE image of comparison between (a) fresh Cu30MnFe, (b) used Cu30MnFe, (c) fresh Cu30MnFekao7.5, and (d) used Cu30MnFekao7.5 external morphology.

In Figure S2 a uniform distribution of three metal oxides of the oxygen carrier in the fresh Cu30MnFe sample is observed.

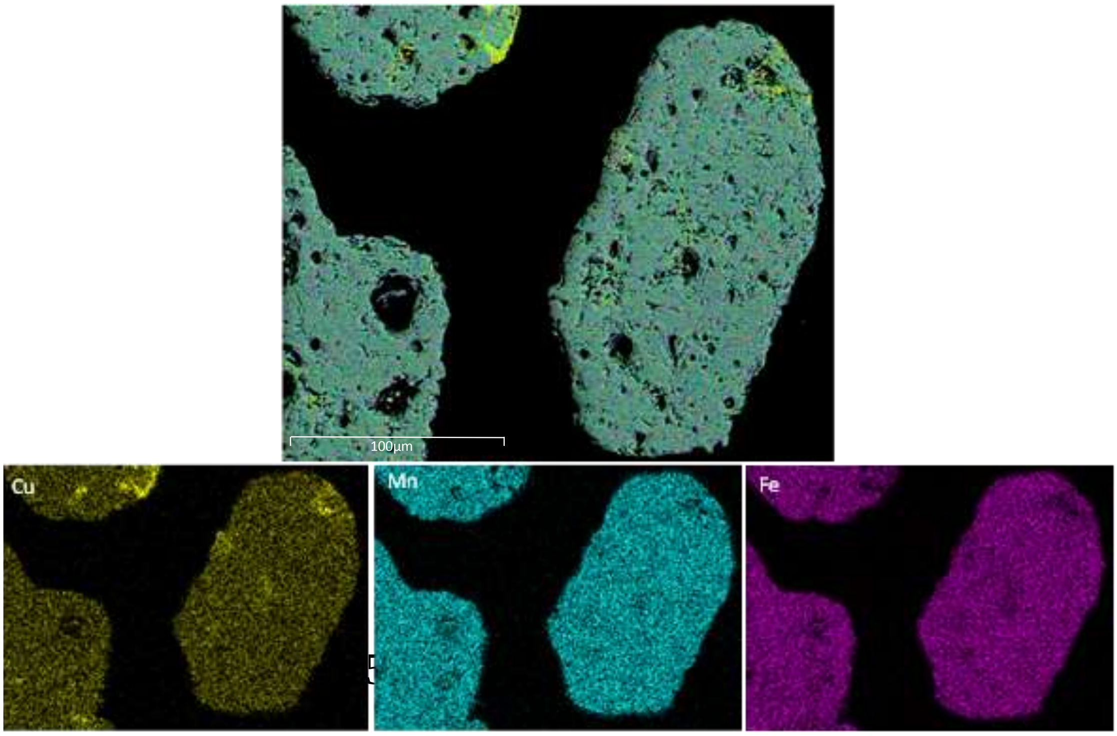

Figure S2. SEM-EDX mapping of fresh Cu30MnFe

Figure S3 depict the distribution of Cu oxide, Mn oxide, and Fe oxide, which remained unchanged during combustion in the plant. Therefore, there was no interaction between the oxygen carrier Cu30MnFe and biomass ash.

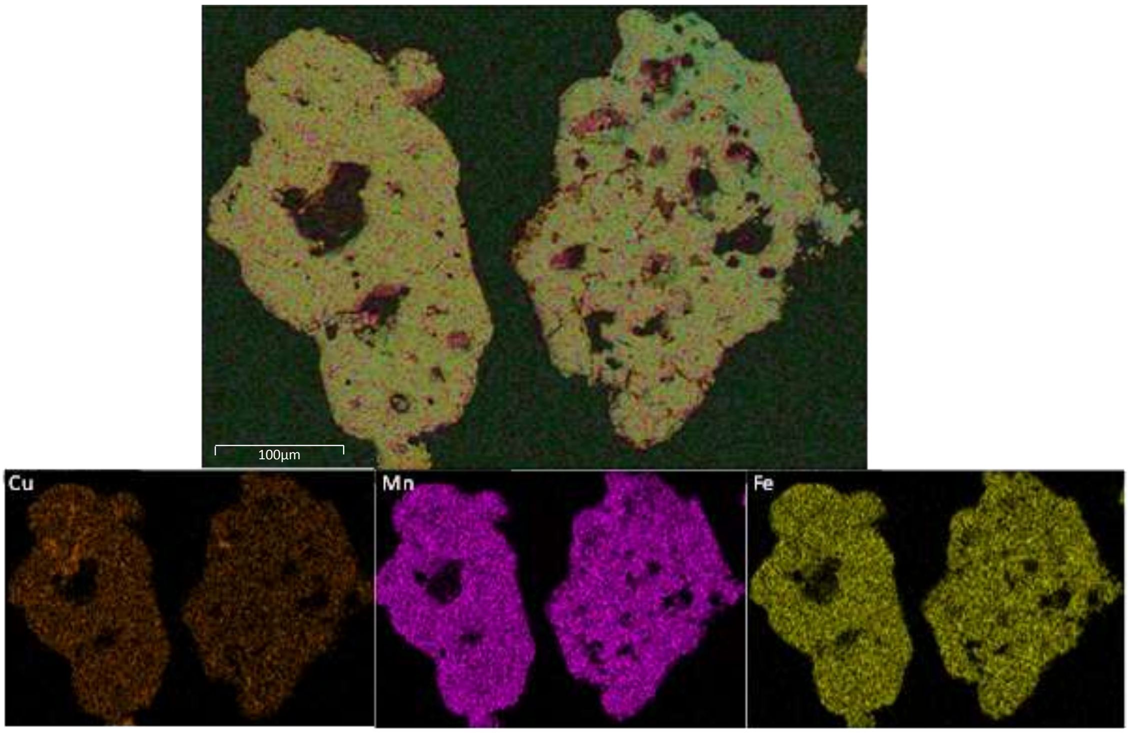

Figure S3. SEM-EDX mapping of used Cu30MnFe.

Figure S4 presents SEM-EDX elemental mapping of the fresh Cu30MnFekao7.5. It reveals that Cu oxide, Mn oxide, and Fe oxide are homogeneously distributed within the particle, along with kaolin, which includes Al and Si. K, Mg, and Ca are also present throughout the particle, but are not concentrated in any specific areas.

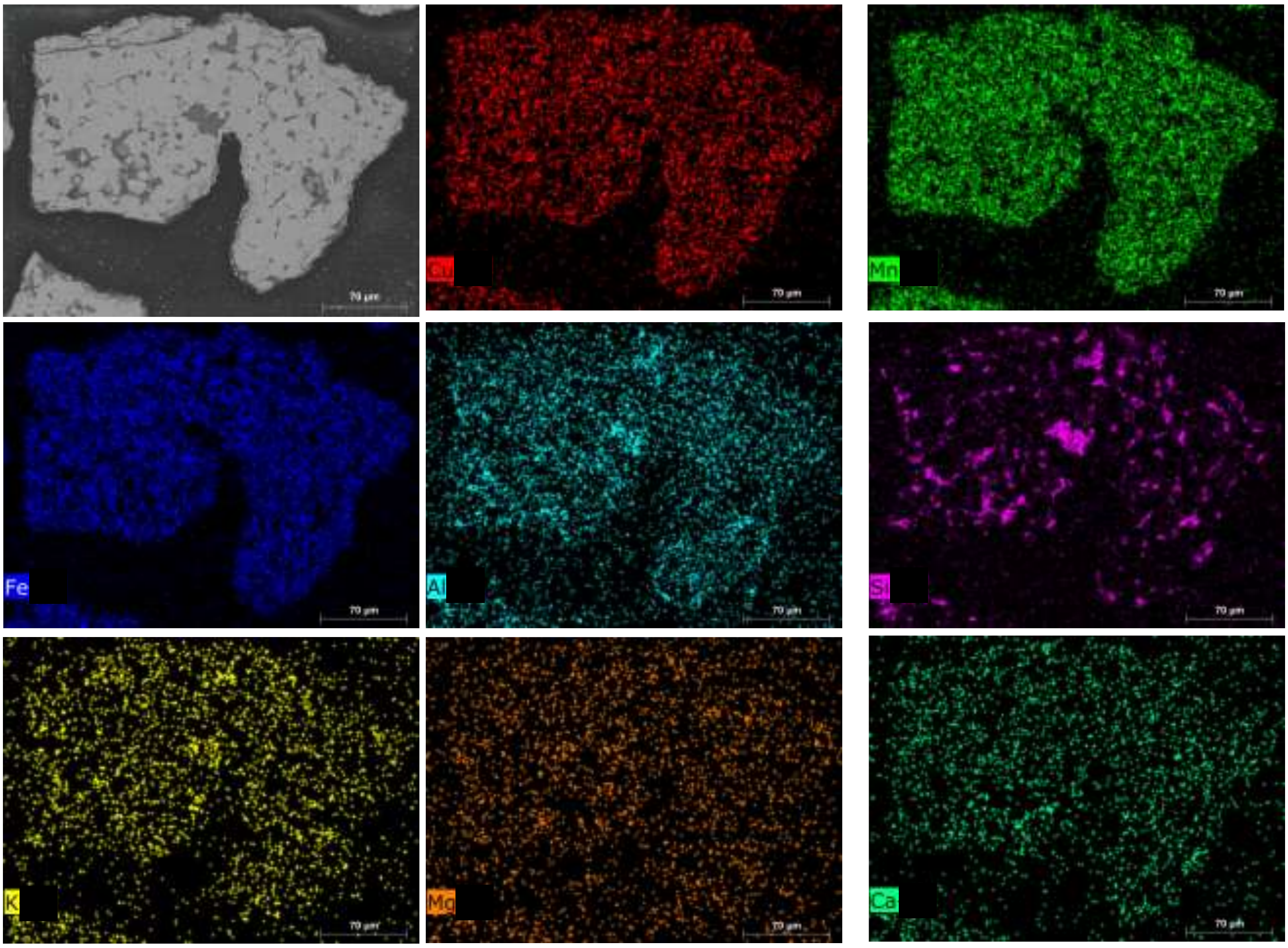

Figure.S4. SEM-EDX elemental mapping of fresh Cu30MnFekao7.5.

Figure S5 presents the SEM-EDX elemental mapping of the used Cu30MnFekao7.5. It shows that K is accumulated in regions containing Al and Si, identified as kaolin. In these areas, the concentration of Cu, Mn, and Fe oxides is low.

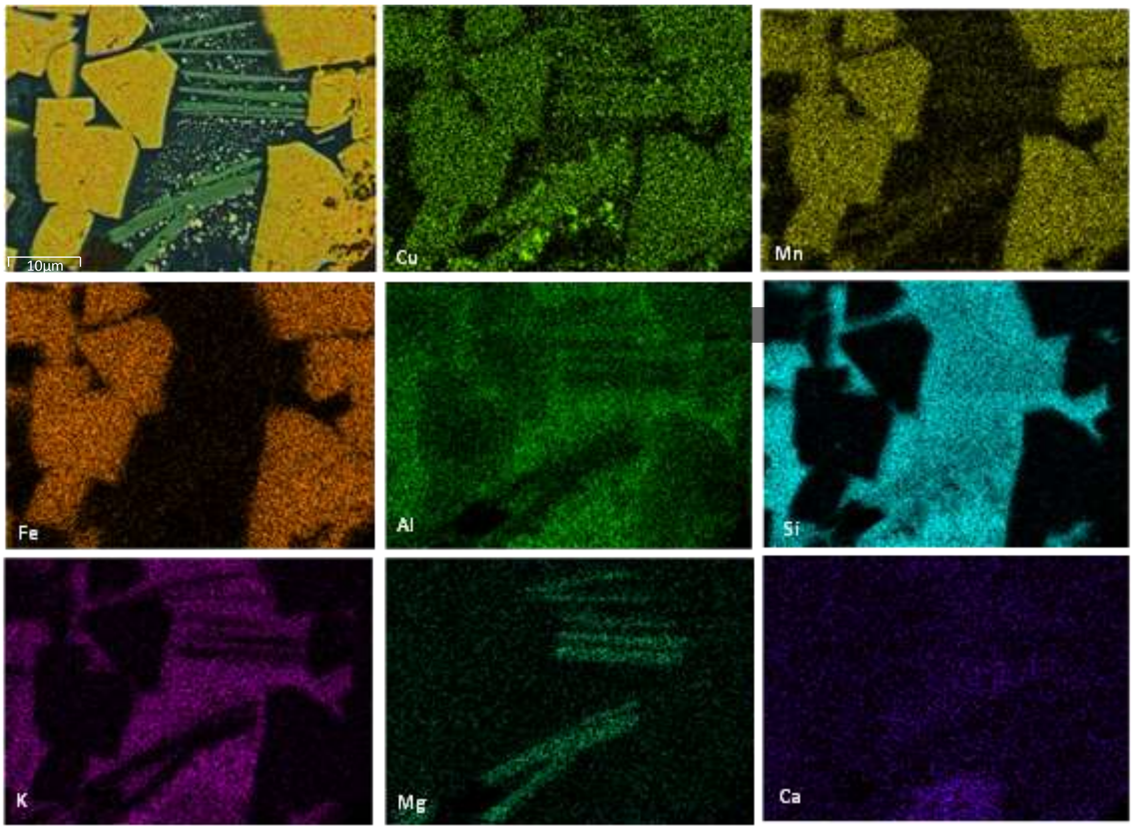

Figure.S5. SEM-EDX elemental mapping of the inner of a used Cu30MnFekao7.5.

Figure S6 presents BSE images of the two particles of Cu30MnFekao7.5 oxygen carrier after 56 hours of combustion. The EDX results reveal that accumulative K can be observed in the darker areas, particularly where there is a high concentration of kaolin. Specifically, at points 13, 15, 16, and 17, where Si and Al are present in the used oxygen carrier Cu30MnFekao7.5, K is also detected. Conversely, at points 14 and 18, where the concentration of Si and Al, indicative of kaolin, are low and the levels of Cu, Mn, and Fe are high, no K is detected. Additionally, surface-section images in Figs. S6b and S6c show that the amount of K on the surface is significantly lower compared to the interior of the particle.

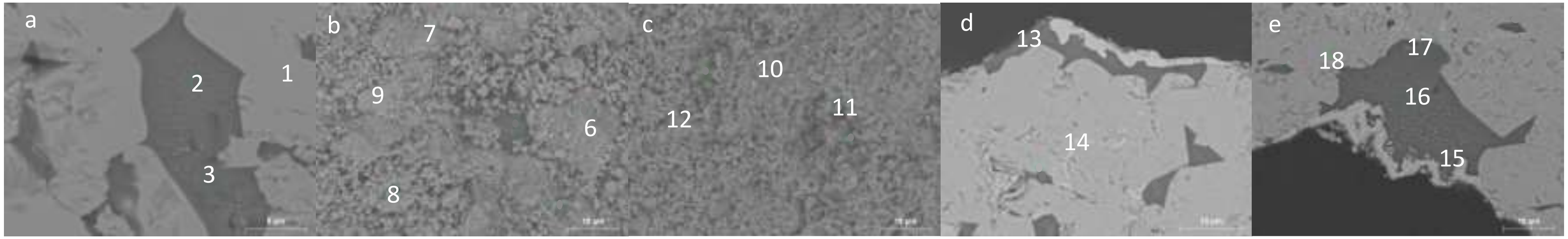

	Cu30MnFekao7.5-Fresh			Cu30MnFekao7.5-Used														
				Surface- section									Cross- section					
Component	Point 1	Point 2	Point 3	Point 4	Point 5	Point 6	Point 7	Point 8	Point 9	Point 10	Point 11	Point 12	Point 13	Point 14	Point 15	Point 16	Point 17	Point 18
Cu (mol%)	10.2	2.7	1.8	5.6	2.9	9.5	8.4	9.4	9.8	6.7	14.4	7.8	3.1	13.2	10.9	2.9	1.7	10.6
Mn (mol%)	11.6	2.1	1.6	5.8	4.7	11.4	10.5	15.8	12.9	9.6	16.0	10.1	2.0	14.2	1.2	1.8	1.7	15.3
Fe (mol%)	13.6	1.9	1.3	7.1	5.3	12.7	10.9	17.4	14.5	10.6	19.2	10.7	1.7	16.3	1.1	1.2	1.4	17.5
Si (mol%)	1.0	18.6	22.5	14.9	15.3	1.0	2.6	5.5	2.7	3.2	2.2	4.6	26	0.9	22.6	25.4	25.1	1.0
Al (mol%)	1.1	3.6	3.0	1.5	1.7	1.1	2.0	1.8	2.3	1.8	1.7	2.1	5.1	1.2	4.5	5.4	3.9	1.3
Mg (mol%)	-	-	-	0.4	0.5	-	0.7	-	-	0.6	-	0.8	-	-	-	-	-	-
K (mol%)	-	1.3	1.8	0.9	0.7	-	0.2	0.4	0.4	0.6	-	0.5	2.95	-	1.9	2.4	2.1	-

Figure S6. SEM-EDX images of (a) cross-section of fresh Cu30MnFekao7.5, (b) surface section of used Cu30MnFekao7.5, (c) surface section of used Cu30MnFekao7.5, (d) cross-section of used Cu30MnFekao7.5, and (e) cross-section of used Cu30MnFekao7.5 after 56 h of pine sawdust combustion, including table of main component changes at different points.

Figure S7 presents the SEM line scan image of the cross-section of the used oxygen carrier Cu30MnFekao7.5. The image reveals that K accumulates in regions where Al and Si are present, which corresponds to kaolin within the particle. In these areas, the concentrations of Cu, Mn, and Fe oxides are low, while the concentrations of Si and Al are high.

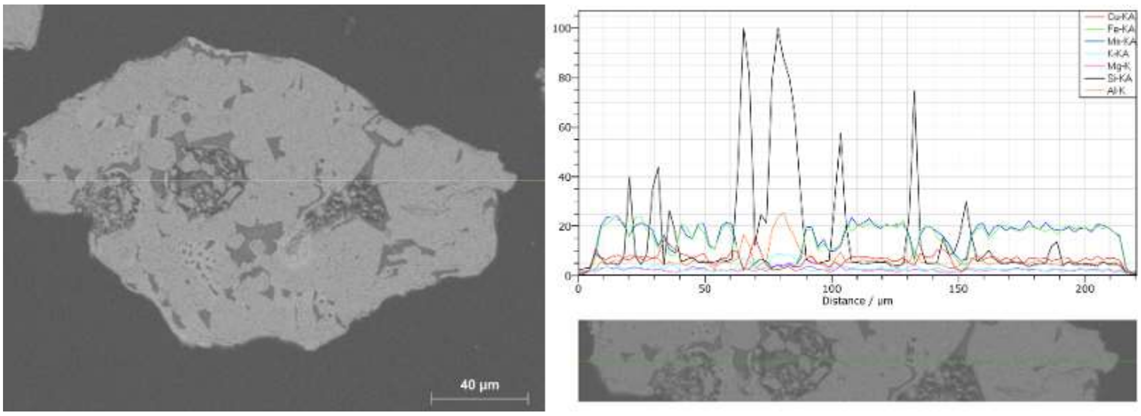

Figure S7. SEM line scan image of cross-section of used Cu30MnFekao7.5 after 56 h of pine sawdust combustion.

Figure S8 presents the SEM-EDX elemental mapping of the surface of the used oxygen carrier, Cu30MnFekao7.5. The analysis indicates the presence of minor amounts of Al and Si on the particle surface, with no significant accumulation of K detected.

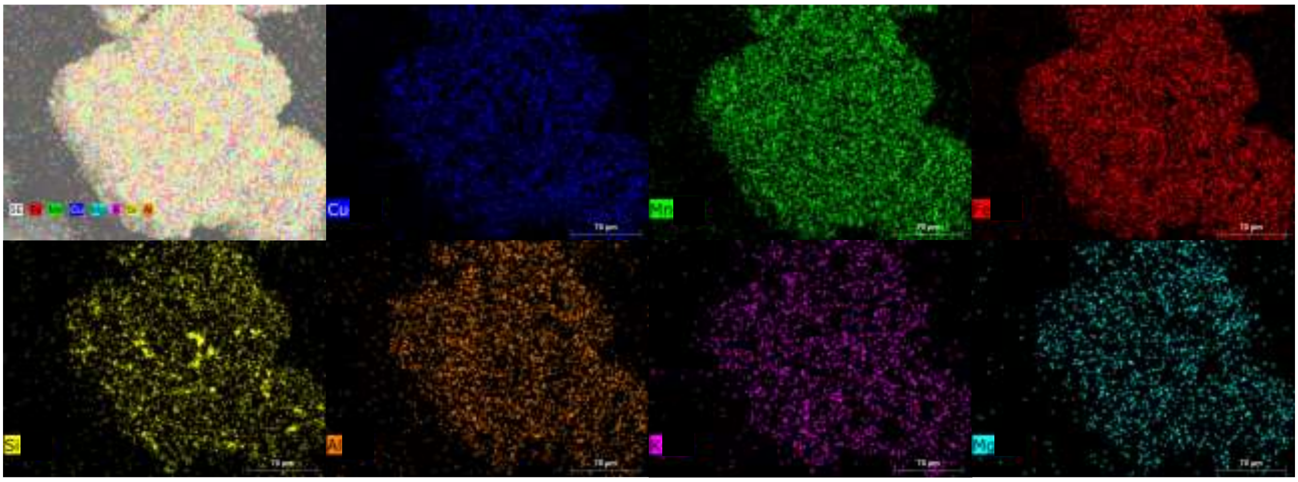

Figure S8. SEM-EDX elemental mapping of the surface of used Cu30MnFekao7.5 following pine sawdust combustion.

Although in SEM-EDX photos for oxygen carrier Cu30MnFe be observed that metal oxides are distributed homogeneously but in Figure S9, free CuO could be found by XRD. It means this free Cu oxide can improve oxygen transport capacity as active phase compared to the fresh oxygen carrier which data in Table 1 confirm this additional potential to release oxygen.

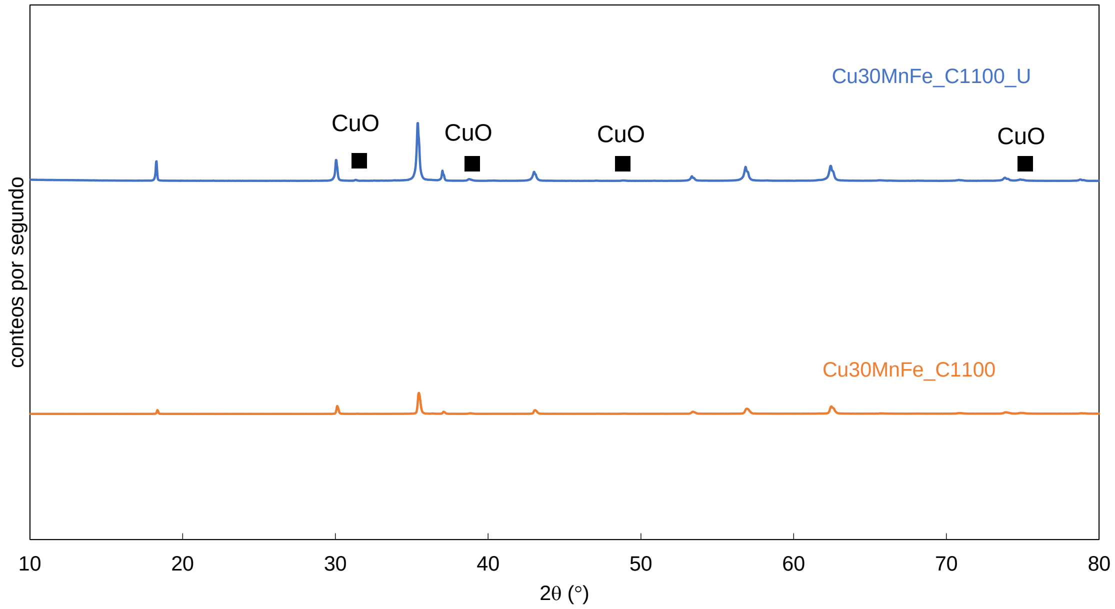

Figure S9. XRD patterns for fresh and used Cu30MnFe
